# Primary knee arthroplasty in Sweden: A nationwide analysis of all‐cause 30‐day mortality and impact of age and ASA‐class

**DOI:** 10.1002/ksa.70102

**Published:** 2025-10-17

**Authors:** Johanna Jarstad, Johanna Albert, Olof Sköldenberg, Jon Karlsson, Jan Gustav Jakobsson

**Affiliations:** ^1^ Department of Clinical Sciences All at Karolinska Institutet, Danderyds Hospital Danderyd Sweden; ^2^ Department of Anaesthesia & Intensive Care Danderyds University Hospital Stockholm Sweden; ^3^ Department of Orthopaedics Danderyds University Hospital Danderyd Sweden; ^4^ Department of Orthopaedics Sahlgrenska University Hospital, Sahlgrenska Academy Gothenburg Sweden

**Keywords:** age, ASA‐class, knee arthroplasty, mortality, perioperative mortality rate (POMR), primary

## Abstract

**Purpose:**

Knee arthroplasty is one of the most common orthopaedic procedures in Sweden, which is in line with many other countries in the Western world. There is no recent data on all‐cause 30‐day mortality associated with primary knee arthroplasty in Sweden during recent years. The purpose of the present observational quality register study was to investigate the mortality at 0, 7, 30 and 90 days after primary knee arthroplasty, and the impact of age and ASA‐class.

**Material:**

Data from SPOR (Swedish PeriOperative Registry) on primary knee arthroplasty (ICD code NGB) registered between 2017 and 2021 in patients ≥18 years was analysed. Perioperative mortality rates and impact of age and ASA were analysed and are presented as numbers, proportions and 95% confidence interval.

**Results:**

The study cohort included 35,794 patients. The study found overall low 30‐day mortality rate, 16 out of 35,794 patients (0.045%, 95% confidence Interval 0.027%–0.071%), no patients died on the day of surgery, six died within one week, while 50 died within 90 days, with no significant differences over the years. High age and higher ASA‐class were associated with higher mortality.

**Conclusions:**

The study showed a low and stable 30‐day mortality associated with primary knee arthroplasty at 0.045%. Age above 80 years and ASA‐Classes III and IV were associated with increased mortality rate, the benefit versus risk in relation to knee arthroplasty procedure should be assessed individually in patients above 80 years and ASA‐Classes III and IV.

**Level of Evidence:**

N/A.

**Abbreviations**
ACS‐NSQIP: American College of Surgeons National Surgical Quality Improvement Program ASA class‐ American Society of Anesthesiology physical status classification
ASA: American Society of Anesthesiology
CI: confidence interval
ICD code: International Classification of Diseases code
NS: non‐significant
POMR: perioperative mortality rate
PONV: postoperative nausea and vomiting
SD: standard deviation
SPOR: Svenskt Peripoerativt Register, Swedish Perioperative Registry

## INTRODUCTION

Knee arthroplasty is one of the most frequently performed orthopaedic procedures in Sweden as well as in many other Western countries as well [1, 12, 13]. Thirty‐day mortality following surgery is a well‐accepted measure of healthcare performance and quality [4, 15]. Studies published during last 10 years' haves shown substantial decrease in early mortality associated with knee arthroplasty. Hunt et al. found a decrease in perioperative mortality from 0.37% in 2003 to 0.20% in 2011 [7]. Berstock et al.'s meta‐analysis covering a 10‐year period up to 2016 including mortality data from 15 different countries, showing likewise a substantially decreased mortality associated to knee arthroplasty 0.49%–0.12% [3]. However, merely one study was included from the Scandinavian countries. There is no information about early mortality associated with primary knee arthroplasty in Sweden during recent years. The purpose of the present observational registry‐based study was to assess all‐cause 30‐day mortality associated with primary knee arthroplasty over a 5‐year period, 2017–2021 in Sweden and whether it decreased over time. Further, to assess the impact of age, sex and American Society of Anesthesiology (ASA)‐class.

## MATERIALS AND METHODS

This is a retrospective registry‐based, nation‐wide cohort study, based on data from Svenskt Peripoerativt Register, Swedish Perioperative Registry (SPOR) examining the all‐cause 30‐day mortality associated with knee arthroplasty surgery between 1 January 2017 and 31 December 2021. The time period was limited by the ethical approval. SPOR is a validated national quality register collecting patient information, related to their perioperative care [6]. The registry collects perioperative data from all major hospitals in Sweden, but lack information about medical history, concomitant medications, and complication after discharge from recovery room. The SPOR registry is interlinked with the Swedish death register providing death date, thus providing information about all‐cause perioperative mortality.

The inclusion criteria were procedures registered in SPOR using International Classification of Diseases (ICD) codes NGB (NGB09‐NGB99). Exclusion criteria was secondary procedures (NGC), and age under 18 years.

Included variables were patient characteristics such as sex (male/female), age (years), ASA‐classes (I, II, III and IV), and perioperative variables, such as year and month of surgery, duration of surgery (minutes), recovery room stay (minutes), and all‐cause mortality within 90 days (deceased/alive).

Primary outcome was all‐cause 30‐day mortality as proportion of all patients. The secondary objectives were deceased day of surgery (Day 0), deceased within 7 and 90 days and the impact of sex, age and ASA‐class. The study did further assess impact of sex, age and ASA‐class on the mortality rate and compared the age‐adjusted mortality to official national statistics data on mortality in Sweden. It further assessed changes in the number of procedures over the study period including the Covid‐pandemic period 2020/2021 and differences between seasons.

The Utstein modified definition of perioperative mortality was used for analysis of mortality rate [4].

### Categorisation of data

Sex was categorised as male and female. Age was categorised into, adults (18–65 years), elderly (66–80 years) and geriatric ( > 80 years if age). ASA‐classes were categorised as ASA low (ASA I–II) and ASA high (ASA III–IV). Time of year was categorised into *Winter*, December, January and February, *Spring*, March, April and May, *Summer*, June, July and August, and *Autumn*, September, October and November.

### Statistics

This is an observational national quality register based study, thus no power analysis has been performed. Continuous numerical variables, such as age, are described as mean, and standard deviation (SD). Categorical data, such as sex and mortality rate are presented as numbers and percent (%). Perioperative mortality rate is reported as percentage of deceased divided by the total number of patients and 95% confidence interval (95% CI).

All data was collected in Excel Version 2412 and further analysis were performed using the SPSS version 29.0.1.0.

## RESULTS

**Figure 1:**
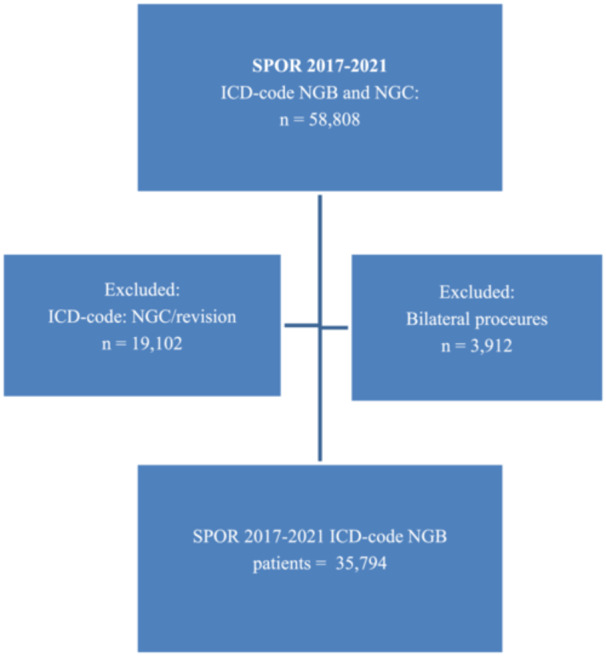
Flow chart of inclusion and exclusion criteria of the study cohort. Presented as number of patients. ICD code, International Classification of Diseases code; SPOR, Svenskt Peripoerativt Register, Swedish Perioperative Registry.

A total of 39,706 knee arthroplasties were performed during the study period, 3912 had both knees operated, thus in all 35,794 patients were included in the analysis set. 20,440 females and 15,317 males (missing information *n* = 37) were included in the analysis. Figure 1 presents the patient inclusion in the study data set.

The number of procedures was highest 2019, while a minor reduction was seen during the Covid‐period 2020–2021 (Table 1). There was also pronounced significant variability in procedures between the months with pronounced drop during summer (Table 2).

**Table 1:** Annual mortality and mortality rate.

	Patients (*n*)	Death (*n*)	% Death at 30‐days	95% CI	
2017	5949	3	0.05%	0.01%	0.13%
2018	7156	4	0.06%	0.02%	0.13%
2019	9730	3	0,03%	0.01%	0.08%
2020	6228	1	0,02%	0.00%	0.08%
2021	6715	5	0,07%	0.03%	0.16%

Abbreviations: CI, confidence interval; *n*, number of patients.

**Table 2:** Mortality for the entire cohort per season.

	Patients (*n*)	Death (*n*)
Winter	10,742	10
Spring	9,166	0
Summer	4,013	2
Autumn	8,609	2

Abbreviation: *n*, number of patients.

### Patients' characteristics and intraoperative variables

Most of the patients were females 20,440 (57%). Mean age for the entire study cohort was 69.6 (SD 9.0) years; identical for females and males. Most patients 22,676 (64.2%) were classified as ASA‐class II, 4723 (13.4%) were ASA‐class I, 7852 (22%) ASA class III and 79 (0.2%) ASA‐class IV (missing information *n* = 464). Patient characteristics showed a variability over the study period, but with no clear increasing or decreasing trend.

Mean duration of surgery, anaesthesia and recovery room stay was, 76 (SD 29), 128 (SD 41) and 310 (SD 150) min, respectively. Cemented total knee arthroplasty was the most common procedure, 28,495 (80%) patients, followed by uncemented total arthroplasty 3378 (9.4%), uncemented partial arthroplasty 2223 (6.2%), cemented partial arthroplasty 1530 (4.3%) and hybrid arthroplasty 79 (0.2%). Information about the surgical technique was missing in 89 patients. Neuraxial anaesthesia was the most common technique, administered in 15,967 patients (70%) while general anaesthesia was used in 7,023 patients (30%). Information on the anaesthetic technique was missing for 12,804 procedures.

### Mortality rate

Among the 35,794 patients included, the all‐cause 30‐day mortality was 16 patients (0.045%; 95% CI: 0.027–0.071%). None of the patients in the cohort died on the day of surgery. In total, six patients died within 7 days, and 50 (0.14%) patients died within 90 days of surgery during the 5‐year study period. Table 3 present 30‐day mortality rate related to sex, age and ASA‐class and 95% CI. There was overall low number of deceased patients, but numerical increase for higher age‐ and ASA‐class. There was a higher proportion of males, who deceased with 30‐days.

**Table 3:** Mortality and mortality rate in relation to sex, low and high ASA‐class and the 3 age‐classes studied.

	Patients (*n*)	Death (*n*)	% Death at 30‐days	95% CI	
Female	20,433	7	0.034%	0.015%	0.067%
Male	15,308	9	0.059%	0.029%	0.107%
Low ASA	27,392	7	0.026%	0.011%	0.050%
High ASA	7922	9	0.113%	0.056%	0.207%
Adults (18–65)	11,137	2	0.018%	0.004%	0.058%
Elderly (66–80)	20,993	10	0.048%	0.024%	0.084%
Geriatric (>80)	3648	4	0.110%	0.037%	0.260%

Abbreviations: ASA American Society for Anesthesiology physical status class; CI confidence interval; *n*, number of patients.

The combined impact of high age and ASA‐class is presented in Table 4. Overall low number of death and elderly and geriatric patients with ASA‐Classes II and IV was expectedly associated with a numerical higher proportion of deceased.

**Table 4:** Mortality and mortality rate in relation to combined high/low ASA‐class and the 3 age‐classes studied.

		Patients (*n*)	Death (*n*)	% Death at 30‐days	95% CI	
Low ASA	Adults (18–65)	9443	1	0.01%	0.00%	0.05%
	Elderly (66–80)	15,721	4	0.03%	001%	0.06%
	Geriatric (>80)	2228	2	0.09%	0.02%	0.29%
High ASA	Adults (18–65)	1544	1	0.07%	0.01%	0.30%
	Elderly (66–80)	5003	6	0.12%	0.05%	0.25%
	Geriatric (>80)	1375	2	0.15%	0.03%	0.47%

Abbreviations: ASA American Society for Anesthesiology physical status class; CI confidence interval; *n*, number of patients.

The annual mortality rate is presented in Table 1. The overall number of deceased was low, and a decrease was seen 2019 and 2020. There was no further reduction 2021. The overall number of procedures did decrease during the Covid‐pandemic years 2020 and 2021.

The number of patients who underwent knee arthroplasty surgery varied between seasons, the highest number was seen during the winter months and lowest during the summer season (Table 2).

The 30‐day mortality rate per decade for the entire cohort is presented in relation to the official national death statistics is presented in Table 5. There was no within 30‐day death in any patient age below 50 years. The mortality rate increase from 50 and was numerically highest 0.112% among patients aged 81–90 years. There was no death within 30‐days among the 73 patients aged between 91 and 100 years that underwent a primary knee arthroplasty. The proportions of patients deceased within 30 days from surgery is numerically in line with the national age adjusted death rate.

**Table 5:** Mortality and mortality rate per decade in relation to official national statistics.

Age class	Patients (*n*)	Death (*n*)	% Death at 30‐days	95% CI	SCB Mortality rate
18–30	24	0		.	*0.003%*
31–40	60	0		.	*0.004%*
41–50	766	0		.	*0.008%*
51–60	5024	1	0.02%	0.002%–0.093%	*0.021%*
61–70	11,857	3	0.025%	0.007%–0.068%	*0.057%*
71–80	14,411	8	0.056%	0.026%–0.105%	*0.17%*
81–90	3579	4	0.112%	0.038%–0.266%	*0.47%*
91–100	73	0		.	*1.5%*
	35,794				

Abbreviations: CI, confidence interval; *n*, number of patients; SCB, Official National Statistics [10].

## DISCUSSION

This observational study based on the Swedish perioperative quality register SPOR data found that early mortality rate associated with primary knee arthroplasty was overall unchanged but far lower (0.045%) than what has been reported in previous studies. Male sex, age above 65 and ASA‐Classes III and IV were the factors associated with higher proportion of deceased within 30‐days. No patient died at the day of surgery and accumulated 50 patients deceased within 90 days (0.14%). The 30‐day mortality rate is in line with the overall age‐adjusted mortality rate in the general population as defined by the official National Statistics [10].

### POMR

The overall all‐cause 30‐day mortality in the present study was lower than what has been reported in previous studies. Berstock et al. meta‐analysis from 2018 showed a 30‐day mortality associated with knee arthroplasty was 0.20% (95% CI 0.17%–0.24%) [3]. A more recent meta‐analysis published in 2023 reported a pooled 30‐day mortality of 0.14% (95% CI: 0.05%–0.22%), which is still almost three times higher than the rate observed in the present study [11]. Also, the 45‐day mortality rate reported by Hunt et al. from the England and Wales registry analysis is clearly higher than the 0.045% found in the present study [7]. Zhai et al. published a study, covering the period 2011–2018 based on the American College of Surgeons National Surgical Quality Improvement Program (ACS‐NSQIP) database showing an overall 30‐day mortality of 0.098% [16]. Sullivan et al. published an update of complications and mortality based on ACS‐NSQIP numbers between 2014 and 2020, showing a 30‐day mortality associated with total knee arthroplasty had decreased to 0.09 [14]. These numbers are more in line with the present findings. Mortality rates in the present study, when data was analysed by age in decades, were similar to what is reported by the official National Statistics of age‐adjusted overall mortality in Sweden [10].

The annual mortality rate was overall stable over the study period, with the highest mortality during the Covid‐year 2021. The present study shows, however, a season variability in mortality with merely four deaths during spring, summer and autumn for the entire study period.

The 30‐day mortality rate in the present study for knee arthroplasty is far lower than what was observed in a study based on SPOR data covering 2013–2022 on primary hip as well as revision arthroplasties [5, 8].

### Strengths and limitations

This study based on the Swedish national quality registry collected data. It covers a 5‐year period. The registry is coordinated with the Swedish death registry collecting date of death from national statistics.

There are, however, also several limitations to this study that must be acknowledged. The registry presents age and ASA‐class, but no further detailed information about comorbidities. Berkovich et al. showed the huge impact on perioperative complications associated with diabetes [2]. There is likewise neither information about the need for transfusion nor other postoperative complications. Maman et al. found in a registry‐based study from Israel no mortality in knee arthroplasty patients without need for blood transfusion and 0.3% in those in need for transfusion [9]. The surgical aspects are likewise not available in the registry and thus not included in the analysis. The overall low number of deceased patients, is indeed reassuring, but limits the possibility for more elaborate statistical analysis. It should also be acknowledged that the period included ended 2021 and further update is warranted.

## CONCLUSION

The Swedish perioperative mortality rate associated with primary knee arthroplasty of 0.045% is low, lower than what has been reported in previous studies and age‐adjusted in line with national age related mortality. The benefit versus risk for knee arthroplasty in patients aged above 80 years and ASA‐classes III and IV should be assessed individually.

## AUTHOR CONTRIBUTIONS

*Concept and protocol*: Jan Jakobsson. *Data handling and analysis*: Johanna Jarstad and Jan Jakobsson (support by Fredrik K Johansson KI statistician). *Writing manuscript*: Johanna Jarstad and Jan Jakobsson. *Manuscript review and input*: Johanna Albert, Olof Sköldenberg, and Jon Karlsson. *Submission*: Jan Jakobsson.

## CONFLICT OF INTEREST STATEMENT

Jan Jakobsson is a paid safety physician for AstraZeneca, no further conflict related to current manuscript.

## ETHICS STATEMENT

None declared.

## Data Availability

Data is available on request from the National Quality Register SPOR.

## References

[1] Arthroplasty for hip and knee. September 4, 2025. Available from: https://www.artrosportalen.lu.se/behandlingar/protesoperation

[2] Berkovich Y , Nissan EC , Maman D , Hirschmann MT , Yonai Y , Steinfeld Y , et al. Diabetes and total knee arthroplasty: a nationwide analysis of complications, hospitalization outcomes and revision burden. Knee Surg Sports Traumatol Arthrosc. 2025;33(9):3250–3260. 40351235 10.1002/ksa.12696 PMC12392386

[3] Berstock JR , Beswick AD , López‐López JA , Whitehouse MR , Blom AW . Mortality after total knee arthroplasty: a systematic review of incidence, temporal trends, and risk factors. J Bone Jt Surg. 2018;100–A(12):1064–1070. 10.2106/JBJS.17.00249 29916935

[4] Davies JI , Gelb AW , Gore‐Booth J , Martin J , Mellin‐Olsen J , Åkerman C , et al. Global surgery, obstetric, and anaesthesia indicator definitions and reporting: an Utstein consensus report. PLoS Med. 2021;18(8):e1003749. 34415914 10.1371/journal.pmed.1003749 PMC8415575

[5] Ekeberg A , Albert J , Sköldenberg O , Karlsson J , Jakobsson JG . 30‐day mortality following revision of hip arthroplasty, a cohort study based on the Swedish Perioperative Registry 2017‐2022. Geriatr Orthop Surg Rehabil. 2025;16:21514593251355915. 40585864 10.1177/21514593251355915 PMC12205194

[6] Holmström B , Enlund G , Spetz P , Frostell C . The Swedish Perioperative Register: description, validation of data mapping and utility. Acta Anaesthesiol Scand. 2023;67(2):233–239. 36424870 10.1111/aas.14174 PMC10108284

[7] Hunt LP , Ben‐Shlomo Y , Clark EM , Dieppe P , Judge A , MacGregor AJ , et al. 45‐day mortality after 467,779 knee replacements for osteoarthritis from the National Joint Registry for England and Wales: an observational study. Lancet. 2014;384(9952):1429–1436. 25012118 10.1016/S0140-6736(14)60540-7

[8] Magnusson J , Karlsson J , Sköldenberg O , Albert J , Frostell C , Jakobsson JG . Difference in early all‐cause mortality among patients having hip arthroplasty a Swedish perioperative registry study 2013‐2022. J Orthop Surg Res. 2024;19(1):295. 38750567 10.1186/s13018-024-04752-6 PMC11094893

[9] Maman D , Nandakumar M , Hirschmann MT , Ofir H , Haddad M , Samir B , et al. Blood transfusion in total knee arthroplasty and total hip arthroplasty: a nationwide study of complications, costs and predictive modelling. J Exp Orthop. 2025;13 12(3):e70317. 40655240 10.1002/jeo2.70317 PMC12255935

[10] National Death Statistic. September 4, 2025. https://www.statistikdatabasen.scb.se/pxweb/sv/ssd/START_BE_BE0101_BE0101I/Dodstal/

[11] Pan X , Turan O , Rullan PJ , Simmons H , Emara AK , Piuzzi NS . 30‐Days to 10‐years mortality rates following total knee arthroplasty: a systematic review and meta‐analysis of the last decade (2011‐2021). J Knee Surg. 2023;36(13):1323–1340. 35901803 10.1055/a-1911-3892

[12] Price AJ , Alvand A , Troelsen A , Katz JN , Hooper G , Gray A , et al. Knee replacement. Lancet. 2018;392(10158):1672–1682. 30496082 10.1016/S0140-6736(18)32344-4

[13] Sahlgrenska University Hospital. Swedish Arthroplasty Register Annual Report, 2022. Available: https://www.sahlgrenska.se/omraden/omrade-3/ortopedi/ledord-susa/svenska-ledprotesregistrets-arsrapport-for-2022-ar-har/

[14] Sullivan GE , Highland KB , Booth GJ , Dunnum AP , Goldman AH . The relationship between age and 30‐day outcomes following unicompartmental versus total knee arthroplasty. J Arthroplasty. 2025;40(3):611–618.e3. 39233099 10.1016/j.arth.2024.08.053

[15] Watters DA , Hollands MJ , Gruen RL , Maoate K , Perndt H , McDougall RJ , et al. Perioperative mortality rate (POMR): a global indicator of access to safe surgery and anaesthesia. World J Surg. 2015;39(4):856–864. 24841805 10.1007/s00268-014-2638-4

[16] Zhai K , Orr M , Grits D , Emara AK , Rothfusz CA , Piuzzi NS . Factors affecting 30‐day mortality following primary elective total knee arthroplasty: a database study of 326,157 patients. J Knee Surg. 2021;36:575–583. 34921379 10.1055/s-0041-1740386

